# An Insight into Prognostic Impact of TIPE2 & CD36 Immunohistochemical Expression in Urothelial Carcinoma 

**DOI:** 10.30699/ijp.2024.2029525.3301

**Published:** 2025-01-10

**Authors:** Amira M. Abd El Maged, Nahla M. Badr, Hanan lotfy Mohammed

**Affiliations:** 1 *Pathology Department, Faculty of Medicine, Menoufia University, Shebin El Kom, Egypt*; 2 *Department of pathology, Faculty of Medicine, Zagazig University, Egypt*

**Keywords:** Urothelial carcinoma, TIPE2, CD36, Immunohistochemistry, epithelial-mesenchymal transition, Prognosis

## Abstract

**Background & Objective:**

While there are fast advances in the detection and management of bladder carcinoma, the number of deaths remains high. Therefore, the identification of an effective biomarker predicting tumor progression in cancer bladder patients is a crucial issue. This study aimed to identify TIPE2 and CD36 expressions in cancer bladder and examine their relationship with clinicopathological data and prognosis.

**Methods:**

Using immunohistochemistry, 60 specimens of bladder urothelial cancer (UC) for the expression of TIPE2 and CD36 were studied and compared with the clinicopathologic parameters and survival data. Furthermore, the association between TIPE2 and CD36 expression and Vimentin expression to elucidate the influence of TIPE2 and CD36 on the epithelial-mesenchymal transition (EMT) were investigated in UC.

**Results:**

TIPE2 expression was associated with lower stages and prolonged disease-free survival (DFS) and overall survival (OS). Therefore, TIPE2 may be considered a good indicator of UC prognosis. CD36 immuno-positivity was associated with high tumor grade, stages, shorter OS, and DFS. Therefore, the immune positivity of CD36 may be a poor prognostic marker for UC patients. Furthermore, Vimentin expression was directly correlated with CD36 expression and inversely correlated with TIPE2 expression.

**Conclusion:**

TIPE2 and CD36 may be novel biomarkers for predicting tumor metastasis and prognosis in patients with bladder UC and hold promise as therapeutic targets.

## Introduction

Bladder cancer (BC) ranked eighth in analyzed deaths and fourth in analyzed new cases in men concerning information from the Statistical Annual Report 2022 of the American Cancer Society (1). The most prevalent histopathological variant of bladder cancer is urothelial carcinoma (UC) (2). Metastases are in charge of the great majority of cancer-related mortalities. Therefore, prediction of tumor aggressiveness and metastatic potential is crucial for prognosis. Currently, prognosis primarily relies on the clinicopathological parameters, and existing prognostic markers are inefficient. Consequently, new biomarkers are highly necessary to estimate tumor metastasis and patient outcomes (3). 

Tumor necrosis factor-α-induced protein-8 (TNFAIP8)-like-2 (TIPE2) is a protein of the TNFAIP8 family (4). TIPE2 has been established to be a negative regulator in both innate and adaptive immunity (5). TIPE2 is detected in different specific epithelial tissues such as squamous, glandular epithelium and lymphoid tissues (6). Interestingly, a considerable volume of literature has reported that TIPE2 performs a critical role in tumorigenesis; several types of tumors can be suppressed by TIPE2 expression e.g. hepatocellular, prostatic, and lung cancer (7-9). However, exact mechanisms are still unclear in majority of these studies; TIPE2 has exhibited its power as a new biomarker and therapeutic target in different malignancies. Considering the extensive research in this area, the effect of TIPE2 on the initiation and aggressiveness of bladder UC remains largely unclear (3). 

One of the hallmarks of cancer development is altered fatty acid (FA) metabolism, and a previous study has suggested that suppressing this pathway may be a promising therapeutic target for cancer (10). Cluster of differentiation 36 (CD36) is one of the FA transporters that has a role in the uptake of long-chain FA (11). Its pathophysiological roles include immune recognition, lipid uptake, inflammation, angiogenesis, molecular adhesion, phagocytosis, apoptosis, and tumor metastasis (12-14). Previous researchers have considered expression of CD36 as an unfavorable factor for prognosis in a variety of tumors (15, 16). Behind the fact that CD36 enhances cancer progression through increasing uptake of FA, it also suppresses neovascularization by interaction with TSP-1 (17, 18). Furthermore, CD36 reprograms the tumor-immune cell's functions resulting in tumor immune tolerance making CD36 a potential target for cancer therapy (19). 

It is known that metastasis accelerates the cancer death rate; it can take place early or late after primary tumor development. Epithelialmesenchymal transition (EMT) is the cardinal step by which epithelial cells gain features of mesenchymal cells. Apart from its role in the normal development of tissue, it is also a pivotal process included in cancer progression and metastasis. Different indicators are involved in controlling EMT, like the elevation of mesenchymal markers expression e.g. vimentin, snail, slug, and twist (20).

CD36 has already been established to enhance EMT in multiple cancers. In the cancer cervix, CD36 enhances EMT via interconnections with the TGF pathway (21). Furthermore, CD36 expression enhances the metastatic activity in colorectal cancer via MMP28 upregulation and loss of E-cadherin (22). On the other hand, prior studies have demonstrated that TIPE2 inhibits the EMT in other cancers. In gastric cancer, overexpression of TIPE2 suppresses metastasis through advancing β-catenin degradation, and in cancer esophagus through blockage of the Wnt/β-catenin pathway (23, 24). In addition, Formal searches have stated that TIPE2 may reverse the EMT process leading to the suppression of the metastatic ability of cancer endometrium through direct binding to β-catenin (25).

Therefore, focus of the present research was to explore the relationship between immunohistochemical expression of TIPE2 and CD36 in urothelial carcinoma and clinicopathological findings and prognosis. Additionally, it elucidated whether this effect works through the EMT pathway.

## Material and Methods

This work was a cross-sectional study that included sixty sections from formalin-fixed paraffin-settled tissue blocks diagnosed as primary bladder carcinoma. The cases were provided retrospectively from the Pathology Department archives, Faculty of Medicine, Menoufia University between January 2020 and December 2022. Clinicopathological information, histopathological reports, and slides stained with hematoxylin and eosin (H&E) for the cases were appraised to ensure an accurate diagnosis. Exclusion criteria include cases with highly necrotic tumors; and cases lacking agreement on histologic diagnosis, grading, or IHC marker scoring. This work was conducted in agreement with the Helsinki Declaration and written consent was taken from each participant. Overall survival data for bladder carcinoma cases were collected by reviewing the patient files from January 2020 and December 2022. The ethical committee of our university (following the Egyptian Ethical Guidelines) approved the protocol for this study.

### Immunohistochemistry

The Streptavidin-biotin amplified system was the method utilized for immunostaining. The slides underwent a consecutive process of deparaffinization, rehydration, and endogenous peroxidase activity blockage. Antigen recovery was achieved by boiling in citrate buffer saline (pH= 6) followed by cooling at room temperature. The slides were then stained with primary antibodies against CD36 (Rabbit monoclonal Ab, Catalog # EPR6573, 1/100 dilution, Abcam, Cambridge, UK), TIPE2 (Polyclonal Antibody, Catalog # PA5-38711, 1:100 dilution, Thermo Scientific, USA), and Vimentin (Polyclonal Antibody, Catalog # PA5-27231, AB_2544707, 1:100 dilution, Thermo Scientific, USA). The primary antibody was brooded overnight at room temperature, followed by the application of a secondary antibody with diaminobenzidine (DAB) as a chromogen substrate and counter-staining with Mayer's hematoxylin.

### Immunostaining Interpretation

Two experienced pathologists independently evaluated and scored the immunohistochemical staining in a blinded manner. A third pathologist was asked to interpret the results and make the final decision when there were inconsistencies. 

The subsequent IHC scoring system was used for evaluation. The intensity of IHC staining was scored as follows: 0 (negative), 1 (weak), 2 (moderate), or 3 (intense). The extent of staining was scored as 0 (negative), 1 (less than 20% of tumor cells stained), 2 (20–50%), 3 (50–80%), or 4 (greater than 80%).

For TIPE2 immunoreactivity, both cytoplasmic and nuclear immunoreactivity were considered TIPE2-Positive. The TIPE2 score was calculated by multiplying the above two scores resulting in a range from zero to 12. Scores from 0 to 3 were considered negative expression, >3 were considered positive expression, < 7 was defined as low expression, and ≥7 was defined as high expression (3).

For CD36 immunoreactivity, membranous with or without cytoplasmic expression was considered CD36-positive. The CD36 expression score was calculated by summing the results of the above two scores which ranged from zero to 7. A final score >4 was considered positive (26). 

For vimentin immunoreactivity, cytoplasmic, with or without nuclear expression was considered vimentin positive. The vimentin expression score was estimated by multiplying the score of intensity by the extent scoring score. Cases were divided into negative expression when the score was ≤ 1 and positive expression when the score was ≥ 2 (27).

### Statistical Analysis

SPSS 22.0 for Windows (SPSS Inc., Chicago, IL., USA) and MedCalc Windows were used for all statistical analyses. The Mann-Whitney U test compared two groups of non-normally distributed variables, while the Kruskal Wallis H test was utilized for comparisons between more than 2 groups. Fisher's exact test or Pearson's Chi-square test was used to compare percentages of categorical variables. In survival analysis, the Kaplan-Meier method was used to estimate survival curves, and the log-rank test was used to analyze it. All differences were considered statistically significant at a P-value of ≤ 0.05.

## Results

### Patients’ Characteristics

The present study included 60 patients with UC. The patient's age distribution ranged between 48-65 years with a mean age of 56.95±6.071 (Table 1). Five of these 60 patients (8.3%) were in the early stage of the disease. On the other hand, 21 of the UC patients (35%) were in the advanced stage of the disease. The median for the follow-up period was 30 months (range: 18-36 months) and 27 (45%) of the patients were disease-free without relapse during the follow-up period. Recurrence occurred in 33 (55%) of the patients, while 33 (55%) of the patients died during the follow-up period. The clinic-pathological features of the 60 patients with UC are summed up in Table 1.

### Association of TIPE2 and CD36 Immunohistochemical Expression with Clinic-pathological Data

**Fig. 1:**
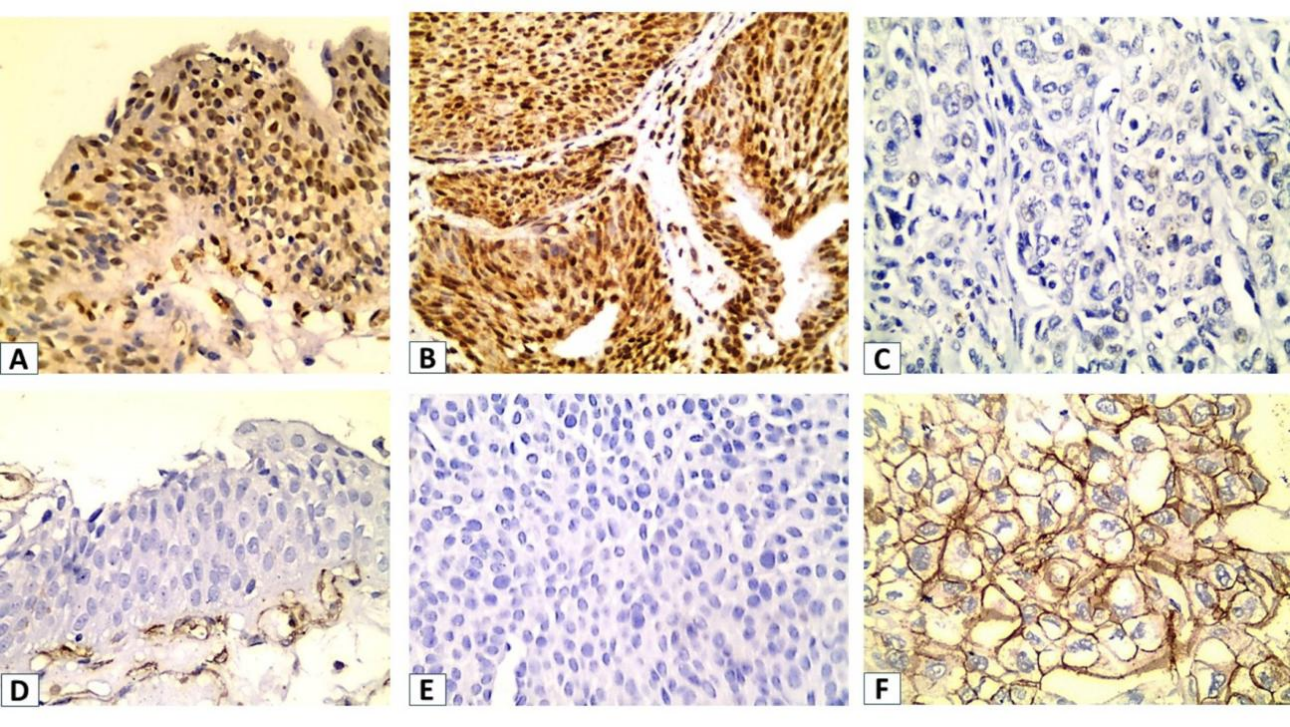
IHC expression of TIPE2 (A: Normal urothelium, B: Low grade UC, C: High grade UC), CD36 Immunoexpression (D: Normal urothelium, E: Low grade UC, F: High grade UC) magnified x 400

**Fig. 2:**
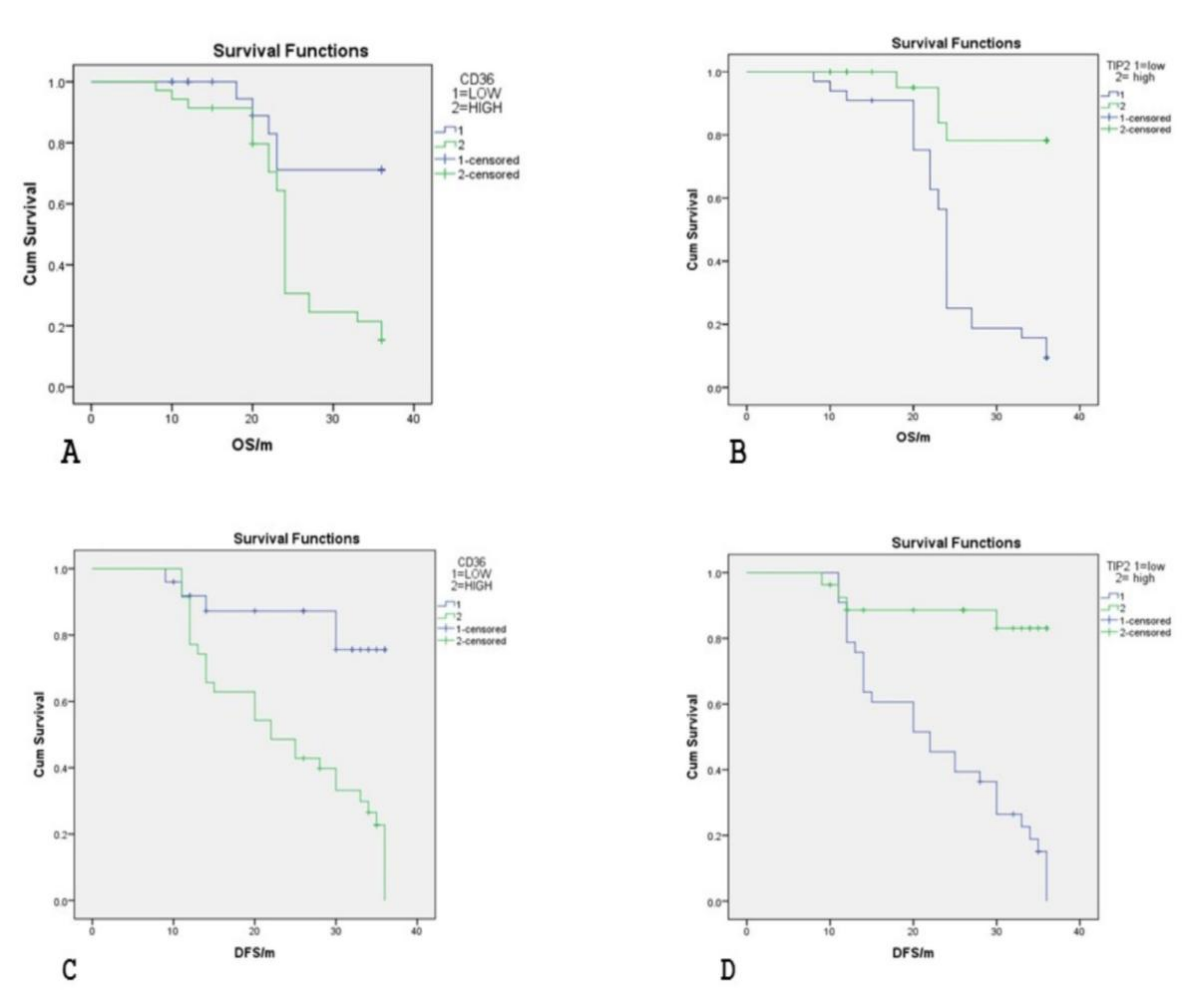
Kaplan Meier plot, Upper panel: Overall survival, lower panel: disease free survival. (A&D) all the studied patient: (A& C) stratified by CD36, (B & D) stratified by TIPE2.

Regarding UC patients, high TIPE2 IHC staining was observed in 25 (41.7%) of the patients, and high CD36 expression was observed in 32 (53.3%) of the patients (Table 1). TIPE2 was stained in the cytoplasm and nucleus of cancer cells (Figure 1), while CD36 was stained in the membrane with or without cytoplasmic expression of cancer cells (Figure 2). 

TIPE2 expression was significantly associated with a lower cancer stage and a low metastatic rate (*P*=0.013 & 0.003, respectively). On the other hand, CD36 expression was correlated significantly with a higher cancer grade and stage (*P*=0.000 & 0.01, respectively). A significant inverse relationship between CD36 and TIPE2 expressions has been detected, in which 23 (71.9%) of CD36 positive cases show low TIPE2 expression, versus 12 (42.9%) of CD36 positive cases showing high TIPE2 immunostaining with (*P*=0.036).

### Association of TIPE2 and CD36 Expression with Survival

Analysis of overall survival (OS) and disease-free survival (DFS) among UC patients using the Kaplan-Meier method (Figure 3) showed that a shorter OS was significantly associated with large tumor size, the presence of distant metastasis, and recurrent disease (*P*=0.028, *P*=0.007, and *P*=0.034, respectively). Conversely, a longer DFS was significantly correlated with a lower cancer stage, absence of distant metastasis, and a low rate of tumor recurrence (*P*=0.04, *P*=0.005, and *P*=0.000, respectively).

Regarding the correlation between the studied biomarkers and OS/DFS, positive TIPE2 expression was associated with longer OS and DFS, while cases positive for CD36 had shorter OS and DFS (Table 3). Thus, CD36 is linked to a poorer prognosis, whereas TIPE2 is associated with a favorable prognosis in UC patients.

### Association of TIPE2 and CD36 Expression with Both Tumor Relapse and Mortality

Concerning the recurrence and mortality rate, low rates of recurrence and patient mortality were associated with high TIPE2 immunostaining, which suggests that the presence of TIPE2-positive immunostaining has a good prognostic impact on UC patients protecting against tumor relapses and mortality. On the other hand, a significant correlation between CD36 positive IHC staining with a high rate of recurrence and mortality was observed, which denotes that CD36 can be considered a poor prognostic indicator (Figure 2, Tables 2).

### Association of TIPE2 and CD36 Immunostaining with Vimentin Expression (Mesenchymal Marker)

As illustrated in Table 4, high CD36 immunostaining was associated with positive Vimentin expression. On the contrary, high TIPE2 immunostaining was associated with negative Vimentin expression; those results suggest that CD36 and TIPE2 may influence UC metastasis through EMT.

## Discussion

 Behavior of the cancer bladder is tricky and unsteady. To date, detection, management, and prognosis of disease rely heavily on imaging examination and invasive cystoscopy with inefficient prognostic indicators in suspecting cancer progression. Consequently, new biomarkers are critically required to increase the accuracy of suspecting tumor aggressiveness and patient outcome.

TIPE2 is a member of the TNFAIP8 family; it not only acts as a negative controller of both innate and adaptive immunity but also has a role in tumor initiation, survival, invasion, and spread (3). TIPE2 has been known as a cancer suppressor in different malignancies such as kidney, colon, and papillary thyroid cancers. TIPE2 has exhibited its powerful role as a new biomarker and therapeutic target in different cancers. However, the action of TIPE2 in the initiation and spread of bladder UC is greatly obscure (28).

In the current work, we investigated IHC expressions of TIPE2 in bladder UC. It was detected that TIPE2 expression was related to low stages of UC and negatively correlated to distant metastasis and relapsing illness in bladder cancer patients. Survival analysis declared its association with both prolonged DFS and OS in UC patients.

These results were consistent with those obtained by Zhu *et al*. (24) who investigated the effect of TIPE2 biomarker in the spread of esophageal carcinomas through Wnt/β-catenin pathway blockage.

Jiang *et al*. (3) stated that TIPE2 could have a crucial effect in the suppression of the initiation and spread of bladder cancer. Their results uncovered the different degrees of TIPE2 expression in bladder cancer tissues, with both cytoplasmic and nuclear location. Furthermore, there was a negative correlation between TIPE2 expression and lymph node invasion and tumor metastases.

Lin *et al*. (29) detected that expression of TIPE2 in gastric cancer (GC) was decreased or even lost and may be associated with initiation of GC. They had put more than one explanation for the inhibitory role of TIPE2 protein in GC, one of these suggestions was that inflammation induced by stimuli such as Helicobacter pylori enhances and promotes apoptosis. TIPE2 plays an important role in this process through the suppression of Bcl-2 and elevate cleaved-caspase-3 and cleaved-caspase-9 levels, which enhance endogenous apoptosis. This might be a pathway to eliminate unwanted cells that have been damaged by reactive oxygen species (ROS) produced by inflammation and to inhibit the survival and proliferation of the damaged cells.

In addition, it was proposed that TIPE2 blocked GC cell proliferation through inhibition of p-ERK and p-AKT, so blocking thus Ras-Raf-MEK-ERK1/2 and PI3K-AKT signaling pathways. Moreover, TIPE2 reduced cyclin B1 and CDK1 expression, thus inducing G2/M-phase arrest. Furthermore, TIPE2 inhibited IL-6, IL-1β, and TNF-α activation decreasing cell proliferation (30).

TIPE2 is involved in tumorigenesis via different signaling pathways, and its precise role in cancers remains to a great extent obscure. Our analysis results concluded that TIPE2 could be considered a tumor suppressor in the initiation and progression of UC and a good indicator for prognosis, which can be used to assess the risk for tumor progression, and as a potential therapeutic target for UC.

Fatty acid (FA) metabolism is an important source of energy for tumor cells; it enhances cancer cell survival, growth, and proliferation by fulfilling energy needs for anabolic processes (31). Moreover, excessive FA oxidation also influences cellular redox conditions by increasing ROS levels. ROS are identified as powerful DNA-damaging factors that not only enhance malignant transformation through increased genomic instability but also enhance cellular proliferation and provide resistance to apoptosis (32).

CD36 is known as a transmembrane receptor with multifunction which is responsible for the uptake of FA. CD36-positive tumors were correlated with poor outcomes, supporting its potential role as a bad prognostic indicator in different malignancies (31). The prognostic effect of CD36 has recently been investigated in many cancers and precancerous cells, and the results declared that the effect of CD36 on cancer relies on the type of cancer (32).

In the current work, we analyzed IHC expression of CD36 in urinary bladder cancer tissue and found that immunopositivity of CD36 was significantly associated with high-grade, stages, greater lymph node invasion, high incidence of tumor recurrence, and higher rates of mortality. Regarding survival analysis, the present study detected that CD36 was associated with shorter OS and DFS.

Several studies have detected a significant correlation between survival and peri-vesical and extra-vesical tissue invasion which has driven prediction that the positivity of CD36 detected in tumors invading the peri-vesical fat might mirror a metabolic change that permits malignant cells to gain the nutritional supplements and energy required to achieve cancer invasion and spread (33).

This work aligns with a study conducted by Jeong *et al*. (2), which explores that high CD36 expression is linked to non-papillary growth type, high grade, high stage, and the presence of metastasis. Jeong *et al*. (26) also stated that CD36 could be utilized as a potential factor in recognizing short OS patients and may be a therapeutic target in UC. In addition, patients with high CD36 levels may require closer monitoring and more aggressive treatment approaches.

Bowden *et al*. (33) investigated cases with non-muscle-invasive high-risk group cancer bladder and revealed a correlation between high CD36 immunohistochemical staining and fast cancer progression. These findings suggest that over-expression of CD36 may be utilized to capture patients with poor outcomes who may benefit from highly extensive lymphadenectomy or a more alternative adjuvant strategy as immunotherapy. 

Our study demonstrated a significant correlation between CD36 immunostaining and shorter both DFS and OS in patients with bladder carcinoma which means that CD36 overexpression was concomitant with tumors displaying highly aggressive behavior. Therefore, patients who test positive for CD36 should be intimately monitored to identify early cancer spread and consider alternative treatments promptly.

Our results give the impression that the immunopositivity of CD36 could be a poor prognostic marker and its IHC analysis may be a beneficial method for detecting the outcome of UC patients. However, the prognostic role of CD36 still needs further investigation.

The primary cause of death is metastasis. One of the initial steps of tumor metastasis is EMT, which directly enhances the invasion and spread of cancer. One of the characteristic features for the development of the EMT phenotype is the elevation of mesenchymal marker expression, e.g. Vimentin, and decreased expression of adhesion markers such as E-cadherin (20).

In this study, our results showed a negative correlation between high TIPE2 expression and positive Vimentin expression. These findings were in agreement with former research, which reported the negative regulatory role of TIPE2 on the EMT process. Wu *et al.* (23) and Zhu *et al.* (24) found that TIPE2 overexpression suppresses gastric and esophageal cancer invasion and metastasis by inhibiting the β-catenin signaling pathway. In addition, Liu *et al.* (25) have shown that TIPE2 may tie to β-catenin directly to switch the development of EMT and inhibit the spread of endometrial cancer. Thus, the findings from the current study support the inhibitory effect of TIPE2 on UC invasion and metastasis by reversing the EMT process.

On the other hand, high CD36 expression directly correlated with positive expression of Vimentin. These results were in agreement with previous studies that identified CD36 as an additional EMT promoter. Deng *et al.* (21) reported that CD36 enhances EMT in cancer cervix via interactions with the TGF- pathway. Drury *et al. *(22) also stated that CD36 expression enhances invasion in CRC via loss of E-cadherin and increase of MMP28 expression. Consequently, our findings supported the emerging links between CD36 expression and UC metastasis through the EMT process.

**Table 1:** Clinico-pathologic parameters of the 60 UC cases

Variable	No (%)
Gender Male Female	42 (70) 18 (30)
Age Mean±SD Median Range	56.95±6.071 57 48-65
Age <50 ≥50	27 (45) 33 (55)
Tumor size (cm) Mean±SD Median Range	4.10±1.458 4 5
Tumor size <3 ≥3	18 (30) 42 (70)
Tumor grade low High	26 (43.3) 34 (56.7)
Tumor stage T1 T2 T3 T4	5 (8.3) 10 (16.7) 24 (40) 21 (35)
Nodal stage N0 N1	15 (25) 45 (75)
Distal metastasis M0 M1	40 (66.7) 20 (33.3)
Relapse Absent present	27 (45) 33 (55)
Death Alive Dead	27 (45) 33 (55)
CD36 expression Low High	28 (46.7) 32 (53.3)
TIPE2 expression Low High	35 (58.3) 25 (41.7)
Vimentin expression Negative Positive	29 (48.3) 31 (51.7)

**Table 2:** Correlation between TIPE2 and CD36 immunostaining with the clinico-pathologic parameters, mortality and relapse

Variable	TIPE2 expression		*Test of Significance* P value	CD 36 Expression		*Test of Significance* P value
	Low expression No (%)	High expression No (%)		Low expression No (%)	High expression No (%)	
Gender Male Female	24 (57.1) 8 (44.4)	18 (42.9) 10 (55.6)	FX=0.3	16 (38.1) 9 (50)	26 (61.9) 9 (50)	FX= 0.4
Age Mean±SD Median Range	58±6 58.5 17	55.75±6.0 57 17	U= 0.23	55.28±5.9 55 17	58.14±5.9 60 17	U= 0.088
Age <50 ≥50	12 (44.4) 20 (60.6)	15 (55.6) 13 (39.4)	FX= 0.16	13 (48.1) 12 (36.4)	14 (51.9) 21 (63.6)	FX= 0.43
Tumor size Mean±SD Median Range	4.41±1.31 4 5	3.75±1.55 4 5	U= 0.05	3.68±1.46 4 5	4.40±1.39 4 5	U= 0.47
Tumor size <3 ≥3	6 (33.3) 26 (61.9)	12 (66.7) 16 (38.1)	FX= 0.05	11 (61.1) 14 (33.3)	7 (38.9) 28 (66.7)	FX= 0.08
UC grade Low High	10 (41.7) 22 (61.1)	14 (58.3) 14 (38.9)	FX= 0.189	18 (75) 7 (19.4)	6 (25) 29 (80.6)	FX= 0.000*
T stage T1 T2 T3 T4	1 (20) 4 (40) 10 (41.7) 17 (81)	4 (80) 6 (60) 14 (58.3) 4 (19)	X= 0.013*	5 (100) 6 (60) 9 (37.5) 5 (23.8)	0 (0) 4 (4) 15 (62.5) 16 (76.2)	X= 0.01*
N stage N0 N1	5 (33.3) 27 (60)	10 (66.7) 18 (40)	FX= 0.134	11 (73.3) 14 (31.1)	4 (26.7) 31 (68.9)	FX= 0.006*
Distal Mets M0 M1	15 (38.5) 17 (81)	24 (61.5) 4 (19)	FX= 0.003*	20 (51.3) 5 (23.8)	19 (48.7) 16 (76.2)	FX= 0.056
Relapse Absent present	7 (26.9) 25 (73.5)	19 (73.1) 9 (26.5)	FX= 0.001*	19 (73.1) 6 (17.6)	7 (26.9) 28 (82.4)	FX= 0.000*
Death Alive Dead	8 (29.6) 24 (72.7)	19(70.4) 9 (27.3)	FX= 0.002*	16 (59.3) 9 (27.3)	11 (40.7) 24 (72.7)	FX= 0.018*
CD36 expression Low High	9 (36) 23 (65.7)	16 (64.0) 12 (34.3)	FX= 0.03*			
TIPE2 expression Low High				9 (28.1) 16 (57.1)	23 (71.9) 12 (42.9)	FX= 0.036*

FX: Fisher’s Exact test

U: Mann Whitney U test

*: significant

**Table 3:** Univariate survival analysis for predicting overall & Disease-free survival in the bladder UC patients

Variable	OS		DFS	
	*HR (CI 95%)*	*P value*	*HR (CI 95%)*	*P value*
Gender Male Female	27.51 (25.03-29.99) 27.52 (23.51-31.52)	0.93	27.21 (24.18-30.22) 25.34 (20.21-30.46)	0.79
Age <50 ≥50	27.92 (24.76-31.08) 27.27 (24.46-30.07)	0.749	26.79 (22.07- 30.88) 26.63 (23.11- 30.16)	0.78
Tumor size <3 ≥3	30.47 (26.42- 34.52) 26.45 (24.12- 2.79)	0.028*	31.39 (26.43- 36.35) 25.52 (22.46- 28.58)	0.12
UC grade Low High	29.29 (25.6-32.99) 26.35 (23.94- 28.76)	0.076	28.93 (24.62- 33.23) 25.73 (22.45- 29.01)	0.058
T stage T1 T2 T3 T4	30.00 (20.39-39.60) 30.05 (24.130-35.97) 28.17 (24.49- 31.84) 25.44 (22.83- 28.04)	0.15	29.8 (20.68- 38.92) 31.0 (23.41- 38.59) 28.26 (24.30-32.22) 23.48 (19.34- 27.61)	0.04*
N stage N0 N1	30.23 (25.38-35.08) 26.63 (24.42-28.84)	0.053	30.86 (24.86-36.87) 25.94 (22.98-28.89)	0.108
Distal metastasis M0 M1	28.94 (25.95- 31.93) 25.44 (22.83- 28.04)	0.007*	28.98 (25.63-32.34) 23.47 (19.34-27.61)	0.005*
Relapse Absent present	33.20 (30.68- 35.72) 23.77 (21.45- 26.08)	0.000*	32.29 (28.93- 35.67) 16.30 (13.69- 18.92)	0.000*
CD36 expression Low High	31.74 (28.56- 34.92) 25.11 (22.63- 27.59)	0.001*	33.43 (30.81- 36.05) 22.15 (18.79- 25.50)	0.000*
TIPE2 expression Low High	24.13 (21.74- 26.54) 32.98 (30.73- 35.12)	0.000*	22.78 (19.51-26.05) 32.78 (29.68- 35.87)	0.000*
Vimentin expression Negative Positive	34.94 (32.92-36.95) 24.10 (21.93- 26.27)	0.000*	34.82 (32.57- 37.07) 22.73 (19.58- 25.87)	0.000*

*: Significant

**Table 4:** Correlation between CD36 and TIPE2 immunostaining with vimentin expression (mesenchymal marker)

Variable	Vimentin expression		*Test of Significance* P value
	Negative expression No (%)	Positive expression No (%)	
CD36 expression Low High	14 (56) 10 (28.6)	11 (44) 25 (71.4)	FX= 0.031*
TIPE2 expression Low High	7 (21.9) 17 (60.7)	25 (78.1) 11 (39.3)	FX= 0.03*

OS: overall survival DFS: disease free survival *: significant
